# Chemoprevention of DMH-Induced Early Colon Carcinogenesis in Male BALB/c Mice by Administration of *Lactobacillus Paracasei* DTA81

**DOI:** 10.3390/microorganisms8121994

**Published:** 2020-12-14

**Authors:** Vinícius da Silva Duarte, Bruna Cristina dos Santos Cruz, Armin Tarrah, Roberto Sousa Dias, Luiza de Paula Dias Moreira, Wilson José Fernandes Lemos Junior, Lívia Carneiro Fidélis Silva, Gabriele Rocha Santana, Leandro Licursi de Oliveira, Maria do Carmo Gouveia Peluzio, Hilario Cuquetto Mantovani, Viviana Corich, Alessio Giacomini, Sérgio Oliveira de Paula

**Affiliations:** 1 Department of Agronomy Food Natural Resources Animals and Environment, University of Padova, Viale dell’Universitá, 16, 35020 Legnaro (PD), Italy; vinicius.dasilvaduarte@unipd.it (V.d.S.D.); tarrah.armin@gmail.com (A.T.); luiza.depauladiasmoreira@studenti.unipd.it (L.d.P.D.M.); viviana.corich@unipd.it (V.C.); 2 Department of Microbiology, Av. Peter Henry Rolfs, s/n, Campus Universitário, Universidade Federal de Viçosa, 36570-900 Vicosa, Brazil; livia.silva@ufv.br (L.C.F.S.); hcm6@ufv.br (H.C.M.); 3 Department of Nutrition and Health, Av. Peter Henry Rolfs, s/n, Campus Universitário, Universidade Federal de Vicosa, 36570-900 Vicosa, Brazil; brunacruz09@yahoo.com.br (B.C.d.S.C.); mcgpeluzio@gmail.com (M.d.C.G.P.); 4 Department of General Biology, Av. Peter Henry Rolfs, s/n, Campus Universitario, Universidade Federal de Vicosa, 36570-900 Vicosa, Brazil; roberto.dias@ufv.br (R.S.D.); gabi.rocha-s@hotmail.com (G.R.S.); leandro.licursi@ufv.br (L.L.d.O.); 5 Faculty of Science and Technology, Free University of Bolzano-Bozen, 39100 Bolzano, Italy; juniorjflemos@gmail.com

**Keywords:** probiotic, colorectal cancer, *Lactobacillus paracasei* DTA81, 1,2-dimethylhydrazine (DMH), short-chain fatty acids, 16S rRNA, oxidative stress biomarkers, cytokine levels

## Abstract

We evaluated the effects of the probiotic candidate *Lactobacillus paracasei* DTA81 (DTA81) on liver oxidative stress, colonic cytokine profile, and gut microbiota in mice with induced early colon carcinogenesis (CRC) by 1,2-dimethylhydrazine (DMH). Animals were divided into four different groups (*n* = 6) and received the following treatments via orogastric gavage for 8 weeks: Group skim milk (GSM): 300 mg/freeze-dried skim milk/day; Group *L. paracasei* DTA81 (DTA81): 3 × 10^9^ colony-forming units (CFU)/day; Group *Lactobacillus rhamnosus* GG (LGG): 3 × 10^9^ CFU/day; Group non-intervention (GNI): 0.1 mL/water/day. A single DMH dose (20 mg/kg body weight) was injected intraperitoneally (i.p), weekly, in all animals (seven applications in total). At the end of the experimental period, DTA81 intake reduced hepatic levels of carbonyl protein and malondialdehyde (MDA). Moreover, low levels of the pro-inflammatory cytokines Interleukin-6 (IL-6) and IL-17, as well as a reduced expression level of the proliferating cell nuclear antigen (PCNA) were observed in colonic homogenates. Lastly, animals who received DTA81 showed an intestinal enrichment of the genus *Ruminiclostridium* and increased concentrations of caecal acetic acid and total short-chain fatty acids. In conclusion, this study indicates that the administration of the probiotic candidate DTA81 can have beneficial effects on the initial stages of CRC development.

## 1. Introduction

Non-communicable diseases (NCDs) account for 63% of global deaths [1]. Among them, colorectal cancer (CRC) is the third most frequently diagnosed worldwide and ranked second in terms of mortality [2]. It is a multifactorial disease, and its increased incidence has been observed in Western countries, particularly in the last few decades. This illness is positively correlated with changes in dietary patterns such as high consumption of ultra-processed food and beverages with low nutritional value, as well as lifestyle factors [3].

Amongst the metabolic changes that precede precancerous cell development, chronic cellular damage due to an imbalance between reactive oxygen and nitrogen species (ROS; RNS) production and antioxidants may lead to DNA damages and genetic mutations [4]. Another factor contributing to CRC development concerns the continuous local inflammatory process, evident in inflammatory bowel disease (IBD), which consists of cellular and humoral responses via increased production of pro-inflammatory cytokines such as IL-6, IL-17, interferon-gamma (IFN-γ), and tumor necrosis factor (TNF-α) in an attempting to regenerate the injured tissue [5,6].

Intrinsically linked to the immune system response pattern and various metabolic processes, the intestinal microbiota and its role in CRC development have been studied in depth in the last few years under health and disease conditions [7,8,9]. As major outcomes, gut microbiome alterations, a process named dysbiosis, along with the identification of CRC-associated microorganisms, have made possible the discovery of diagnostic markers and the potential use of specific bacterial genera for prophylactic and/or therapeutic purposes [10,11].

In this context, several studies have been carried out to identify new chemopreventive agents or functional foods with a recognized beneficial effect towards CRC prevention [12,13,14,15]. In a systematic review conducted by Cruz et al. (2020) [16], the authors emphasize that probiotics and synbiotics can prevent colorectal cancer through different mechanisms such as intestinal microbiota and immune response modulation, reduction of inflammation, biosynthesis of compounds with anticarcinogenic activity, and improvement in redox system balance.

Overall, dairy products are considered a rich and deliverable source of lactic acid bacteria (LAB) with potential probiotic properties [17]. Among the cultures used in the manufacture of fermented dairy, the genus *Lactobacillus* includes a great number of species considered as GRAS (generally recognized as safe) such as *L. rhamnosus*, *L. casei*, *L. paracasei*, *L. plantarum,* and *L. acidophilus* [18].

Based on a recent study conducted by Tarrah et al. (2019) [19], the strain *L. paracasei* DTA81 demonstrated an interesting antiproliferative activity and high adherence ability against the human colorectal adenocarcinoma HT-29 cell line. However, no information is available concerning its prophylactic capability to prevent precancerous lesions during in vivo studies on animal models. Therefore, the present study aimed to evaluate the effects of DTA81 on the reduction of oxidative stress, colonic cytokine profile, caecal short-chain fatty acids (SCFAs) production, and gut microbiota modulation in an animal model of colorectal cancer.

## 2. Materials and Methods

### 2.1. Probiotic Strains, Fermented Milk Production, and Probiotic Dose

*L. paracasei* DTA81 was isolated from a Brazilian infant’s stool [19]. The commercial probiotic strain *L. rhamnosus* GG was included for comparison. *Lactobacillus* strains were routinely grown in De Man, Rogosa and Sharpe (MRS) broth (Oxoid, Milan, Italy) at 37 °C for 24 h aerobically. Fermented milk was prepared following the procedures described by Iyer and Tomar (2011) [20], with some modifications. A total of 100 mL of skim milk was inoculated with each bacterial strain (single batches) at 2% (*v*/*v*) and incubated for 48 h at 37 °C. After this period, another round of fermentation (48 h at 37 °C) was performed by the addition of 100 mL of skim milk. This procedure aimed to increase cellular yield after the fermentation period. Finally, the fermented milk obtained was frozen at −80 °C and freeze-dried to reduce the volume of gavage administered to the animals. After that, freeze-dried fermented milk was produced for each strain, total viable cell counting was performed through plating serial dilutions (1 g sample dissolved in 9 mL phosphate-buffered saline (PBS) pH 7.2 (NaCl 0.13 M, KCl 2 mM, Na_2_HPO_4_ 9 mM, KH_2_PO_4_ 1 mM)) on MRS agar medium. Plates were incubated at 37 °C for 48 h. Results were expressed in colony-forming units (CFU)/g. The probiotics (300 mg) were resuspended in 0.1 mL of autoclaved tap water daily and supplied to their respective group of mice as described below.

### 2.2. Animals and Experimental Design

#### 2.2.1. Animals

Twenty-four male BALB/c mice (*Mus musculus*) at 12 weeks old, were obtained from the Central Animal Facility of the Center for Biological Sciences and Health at Universidade Federal de Vicosa, Minas Gerais, Brazil. In order to maintain homogeneity among the experimental groups, animals were divided according to body weight into four different groups (*n* = 6). The animals were collectively allocated in polypropylene cages with three mice each, kept under controlled conditions, at a temperature of 22 ± 2 °C and humidity of 60–70%, with a 12 h light/dark cycle. The animals received free access to a standard pellet diet (Presence^®^, Paulinia, Brazil) and water throughout the experimental period.

All experimental procedures using animals were performed following Directive 86/609/EEC of 24 November 1986, in compliance with the ethical principles for animal experimentation. The study protocol was approved by the Ethics Committee of the Universidade Federal de Vicosa (CEUA/UFV, protocol n° 15/2020; date of approval: 4 August 2020).

#### 2.2.2. Study Design

After the acclimation period, animals received in the first experimental week via orogastric gavage their respective treatments. From the second week onwards, the protocol for induction of colon carcinogenesis was introduced for all animals enrolled in this study. The colon carcinogen 1,2-dimethylhydrazine (DMH) (Sigma-Aldrich, St. Louis, USA) was prepared in 0.9% saline solution containing 1 mM EDTA and 10 mM sodium citrate, pH 8, as described by Newell and Heddle (2004) [21]. A DMH single dose (20 mg/kg body weight) was injected intraperitoneally (i.p), once a week, performing a total of seven applications per animal in all groups.

The experimental treatments were organized as follows:

Group GSM (skim milk): 300 mg of freeze-dried skim milk resuspended with 0.1 mL of sterile tap water.
Group DTA81 (*L. paracasei* DTA81): 300 mg of freeze-dried fermented milk containing ~3 × 10^9^ cells/0.1 mL of sterile tap water daily.
Group LGG (*L. rhamnosus* GG): 300 mg of freeze-dried fermented milk containing ~3 × 10^9^ cells/0.1 mL of sterile tap water daily.
Group GNI (non-intervention): 0.1 mL of sterile tap water daily.

### 2.3. Food Intake, Body Weight, and Feces Collection

To evaluate the physiological effects of the prophylactic administration of probiotics in weight loss/gain of the animals, food intake and body weight were monitored regularly after the beginning of the DMH protocol. Food intake was measured daily and was calculated by the difference in the amount of diet offered (g) for each group (*n* = 6) and the remaining amount in the day after. At the end of each experimental week, body weight was measured using a digital semi-analytical scale.

The feces of each animal involved in this study were harvested at the beginning (*t*0) and one day before at the end of the experimental period (*t*1). To obtain fecal samples, individual cages were previously cleaned, sanitized, and kept with a single mouse until enough feces were spontaneously expelled. Samples were kept at −80 °C for further analysis.

### 2.4. Euthanasia

After the end of the experimental period (8 weeks), all animals were anesthetized (ketamine 80 mg/kg; xylazine 20 mg/kg) and blood samples were collected from the retro-orbital sinus. The mice were sacrificed by cervical dislocation. The liver and caecum were harvested, snap-frozen in nitrogen liquid, and stored at −80 °C, whereas the colon was resected, washed with cold PBS solution, and sliced into small fragments. These fragments were equally distributed in three different microcentrifuge tubes, with one of them prefilled with RNAlater (Sigma-Aldrich, St. Louis, MI, USA), and stored at −80 °C.

### 2.5. Evaluation of Liver Oxidative Stress Markers

In total, four oxidative stress markers were chosen to assess liver oxidant status. The liver sample was prepared by homogenizing approximately 200 mg of tissue with 800 μL of 50 mM PBS solution containing 1 mM EDTA (pH 7.4). The homogenate was centrifuged at 10,000× *g* for 10 min at 4 °C and the supernatant obtained was used for oxidative markers quantification. For the determination of total proteins in the liver homogenate, the method described by Lowry et al. (1951) [22] was adopted. Catalase (CAT) activity, determined by its ability to cleave hydrogen peroxide (H_2_O_2_) into the water and molecular oxygen, was evaluated according to the method described by Aebi (1984) [23] and the results are expressed as U CAT/mg protein. The superoxide dismutase (SOD) activity, defined as the enzyme amount causing 50% inhibition in pyrogallol autoxidation, was performed according to the methodology described by Marklund (1985) [24] and the results are expressed as U SOD/mg protein. The liver concentration of malondialdehyde (MDA) was determined as thiobarbituric acid reactive substances (TBARS) of lipid peroxidation and followed the methodology described by Buege and Aust (1978) [25]. The results were expressed as nmol of MDA/mg protein. Lastly, the level of liver protein oxidative damage, indicated by the levels of protein carbonyls, was measured according to the method of Levine et al. (1990) [26].

### 2.6. Cytokine Profile in Colon Homogenate

To determine colonic cytokines production, colon samples (100 to 200 mg) were ground using a tissue homogenizer (IKA WORKS GMBH and CO, Staufen, Germany, model T10 basic) in PBS buffer (pH 7.0), centrifuged (10,000× *g*, for 10 min at 4 °C) and the supernatant recovered. Pro- and anti-inflammatory cytokines (Interleukin-2; Interleukin-4, Interleukin-6, Interferon-γ, Tumor Necrosis Factor, Interleukin-17A, and Interleukin-10) were simultaneously determined by the Cytometric Bead Array (CBA) mouse Th1/Th2/Th17 Kit in a BD FACSVerse Flow cytometry following the manufacturer’s recommendations (Becton, Dickinson, and Company, Franklin Lakes, NJ, USA). The data were processed using the FCAP Array Software v3.0 and the results were expressed in pg/g of tissue.

### 2.7. Fecal Short-Chain Fatty Acids (SCFAs) Quantification

The SCFA profile of acetate, propionate, butyrate, and lactate present in the caecum of all the animals was evaluated by high-performance liquid chromatography (HPLC) according to the method described by Siegfried et al. (1984) [27]. Briefly, approximately 100 mg of caecal content were thawed at room temperature, homogenized in 300 µL of distilled water, and centrifuged at 12,000× *g* for 10 min. After that, 300 µL of supernatant was recovered and transferred to a new microcentrifuge tube where 300 µL of calcium hydroxide solution (CHS) and 150 µL of cupric sulphate solution (CSR) were thawed at room temperature and then centrifuged at 12,000× *g* for 10 min. To the supernatant (500 µL), 14 µL of H_2_SO_4_ concentrated was added, and the tubes were frozen. Lastly, tubes were centrifuged (12,000× *g* for 10 min) and 300 µL was recovered and stored in an HPLC vial at 4 °C until the analysis. For the determination of the fatty acids, a Dionex Ultimate 3000 HPLC system (Thermo Scientific, Bremen, Germany) was used. Samples were separated in a RezexROA column–Organic acid H^+^ (8%), (Phenomenex, Torrance, EUA), 300 mm × 7.8 mm at a flow rate of 0.7 mL/min. The mobile phase was H_2_SO_4_ 5 mM. The column temperature was set at 45 °C and the injection volume was 20 µL. Results were expressed in µmol/g feces.

### 2.8. Quantitative Real-Time Polymerase Chain Reaction (RT-qPCR)

The relative expression level of tumor-related genes was evaluated by qPCR in an Illumina Eco^®^ real-time polymerase chain reaction system (Illumina, San Diego, CA, USA), using the GoTaq^®^ 1-Step RT-qPCR System (Promega, Madison, WI, USA). After extraction following Tryzol^®^ reagent (Thermo-Fisher, Waltham, MA, USA) protocol, the RNA was quantified using Qubit 4 Fluorometer (Thermo-Fisher). The thermal cycles used were the default of the equipment: 10 min at 94 °C to polymerase activation, followed by 40 cycles of 10 sec at 95 °C to open strands and 30 sec at 60 °C to annealing and extension. The primers used are listed in Table 1. The analysis was performed by the 2^−ΔCt^ method on the EcoStudy^®^ software (Illumina, San Diego, CA, USA), using GAPDH as an endogenous control.

### 2.9. Fecal Bacterial Composition Analysis Using Next-Generation Sequencing (NGS)

#### 2.9.1. DNA Extraction

Mice fecal samples (250 mg) were obtained before probiotics administration (*t*0) and at the end of the experimental period (*t*1). The metagenomic DNA was extracted using the DNeasy PowerLyzer PowerSoil DNA isolation kit (Qiagen, Hilden, Germany) according to the manufacturer’s instructions. DNA concentration and purity were determined via 260/280 and 260/230 ratios measured on the NanoDrop 2000c (Thermo Fisher Scientific, Waltham, MA, USA).

#### 2.9.2. 16S rRNA Gene Amplicon Sequencing

After stool DNA extraction, samples of four different animals composing each experimental group at the times *t*0 and *t*1 (32 samples) were collected and sent for NGS at Molecular Research DNA (MR DNA, Shallowater, TX, USA). To assess the gut microbial profile, the hypervariable region V1-V2 of the 16S rRNA gene was chosen. Briefly, PCR primers 27f/338r with a barcode on the forward primer were used in a 30-cycle PCR using the HotStarTaq Plus Master Mix Kit (Qiagen, Germantown, MD, USA) under the following conditions: 94 °C for 3 min, followed by 30 cycles of 94 °C for 30 s, 53 °C for 40 s and 72 °C for 1 min, after which a final elongation step at 72 °C for 5 min was performed. After amplification, PCR products were checked in 2% agarose gel to determine the success of amplification and the relative intensity of bands. Multiple samples were pooled together in equal proportions based on their molecular weight and DNA concentrations, purified using calibrated Ampure XP beads, and then used to prepare Illumina DNA library. Sequencing was performed on a MiSeq (Illumina, San Diego, CA, USA) following the manufacturer’s guidelines generating 300 bp paired-end (PE) reads.

#### 2.9.3. Bioinformatic Data Analyses

Sequence data were processed using the Molecular Research DNA analysis pipeline (MR DNA). In summary, sequences were joined, depleted of barcodes then sequences <150 bp removed, sequences with ambiguous base calls removed. Sequences were denoised, operational taxonomic units (OTUs) generated and chimeras removed. OTUs were defined by clustering at a 97% similarity. Final OTUs were taxonomically classified using BLASTn against a curated database derived from RDPII and NCBI. Raw reads were deposited in the Sequence Read Archive (SRA) database under the BioProject PRJNA661570.

The file containing the OTU abundance information, the metadata of the experimental design, and its phylogenetic tree were imported into the MicrobiomeAnalyst web-based application [33], where statistical analyses were carried out except for alpha diversity comparison, conducted with GraphPad Prism 7 (see Item 2.10). Following data inspection, removal of low count/variance sequences, and data scaling (cumulative sum scaling–CSS) were conducted with default parameters. When appropriate, data were rarefied to the minimum library size (26,070 sequences).

Differences between gut microbial community structure (beta diversity analysis) before and after the experimental period were calculated by permutational multivariate analysis of variance (PERMANOVA). Scatter plots of the principal coordinate analysis (PCoA) were computed from the calculated unweighted and weighted UniFrac distance matrixes.

Lastly, the significant difference of the most abundant OTUs among the groups at different times was assessed using the linear discriminant analysis (LDA) effect size (LEfSe) [34] tool setting up alpha-value of 0.05 and Log LDA threshold of 2.0.

### 2.10. Statistical Analysis

Variables were checked for Gaussian distribution with the Shapiro–Wilk test. The intergroup variation was assessed by one-way analysis of variance (ANOVA) using Graphpad Prism Version 7.00 (San Diego, CA, USA). In all significant results, post hoc comparison was performed using Tukey’s multiple comparisons test. For those variables with non-normal distribution, the non-parametric Kruskal–Wallis method was adopted with Dunn’s as a post hoc multiple comparisons test. Differences were considered significant at *p* < 0.05. Unpaired *t*-test with Welch’s correction was used to evaluate animal food intake before and after DMH injection. All the results were expressed as mean ± standard error of the mean (SEM).

## 3. Results

### 3.1. Food Intake and Body Weight

**Figure 1:**
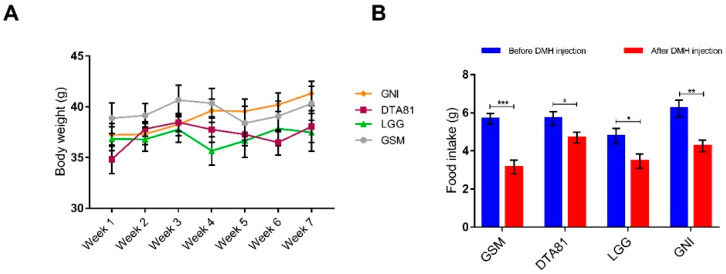
(**A**) Changes in BALB/c mice body weight during the experimental period in the control (skim milk, GSM, and non-intervention, GNI) and probiotic (*L. paracasei* DTA81, and *L. rhamnosus* GG, LGG) groups. (**B**) Food intake was significantly reduced in all groups following 1,2-dimethylhydrazine (DMH) injection. Data represent the mean weight during the experimental period. Data are shown as mean ± standard error of the mean (SEM, *n* = 6). *, *p* ≤ 0.05; **, *p* ≤ 0.01; ***, *p* ≤ 0.001.

Four experimental groups were formed and received a commercial standard pellet diet during the entire experimental period. One group (LGG) received the strain *L. rhamnosus* GG and another group (DTA81) received the strain *L. paracasei* DTA81, both dispersed in skim milk. One group (GSM) received skim milk without bacteria and the last one neither bacteria nor skim milk. All groups were treated with DMH to induce pre-neoplastic lesions. All groups started the experimental period with homogeneous body weight (Figure S1) and there were no significant differences (*p* > 0.05) in body weight among all groups (Figure 1A). In terms of food intake, there were no significant differences among the groups neither before nor after DHM injection (Figure S2). However, a significant reduction in food consumption (intra-group comparison) on the day after DMH injection was observed in all experimental groups (Figure 1B).

### 3.2. Effect of L. Paracasei DTA81 on Oxidative Stress Biomarkers in the Liver

**Figure 2:**
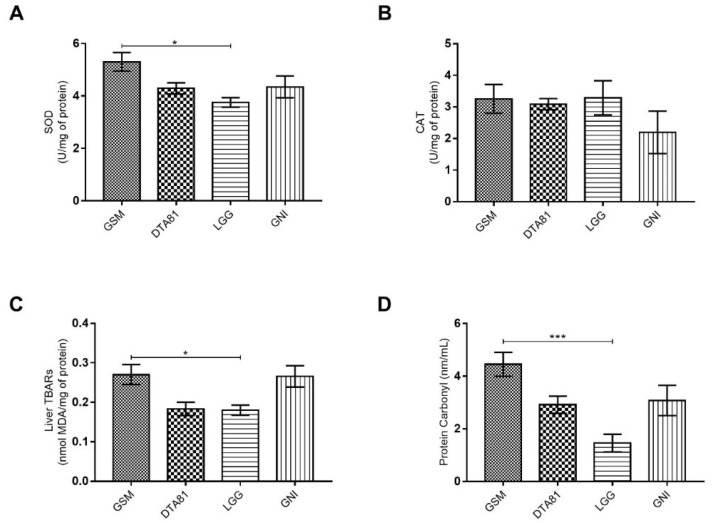
Oxidant status markers in the liver. Effect of *L. paracasei* DTA81 (DTA81) or *L. rhamnosus* GG (LGG) in liver tissue of male BALB/c animals subjected to 1,2-dimethylhydrazine. (**A**) superoxide dismutase (SOD) activity; (**B**) catalase (CAT) activity; (**C**) malondialdehyde (MDA); (**D**) protein carbonyls. The data are expressed as means ± SEM (*n* = 6). *, *p* ≤ 0.05; ***, *p* ≤ 0.001.

The effects of supplementation of strains *L. paracasei* DTA81 and *L. rhamnosus* GG on biomarkers of oxidative stress were evaluated by measuring the enzymes involved in the endogenous antioxidant defense system (Superoxide Dismutase–SOD; and Catalase–CAT), along with the products of lipid and protein oxidation, MDA and protein carbonyl, respectively. The daily intake of LGG significantly decreased the hepatic level of SOD only when compared to the experimental group that received skim milk (group GSM) (Figure 2A), however, there was no significant difference among the experimental groups concerning CAT levels (Figure 2B). The determination of MDA levels in mouse liver (Figure 2C) revealed that the animals fed with the strains *L. rhamnosus* GG and *L. paracasei* DTA81 showed on average reduced levels of lipid peroxidation in liver homogenate when compared to GSM and GNI groups, although statistical significance has been observed only between LGG and GSM (Figure 2C–group GSM: mean = 0.27, coefficient of variation = 21.10%; group DTA81: mean = 0.18, coefficient of variation = 20.89%; group LGG: mean = 0.18, coefficient of variation = 15.82%; group GNI: mean = 0.26, coefficient of variation = 24.73%). Lastly, animals supplemented with LGG had a significant decrease level of carbonyl proteins when compared to the group fed with skim milk (Figure 2D).

### 3.3. Cytokine Production Profile in Colon Tissue

**Figure 3:**
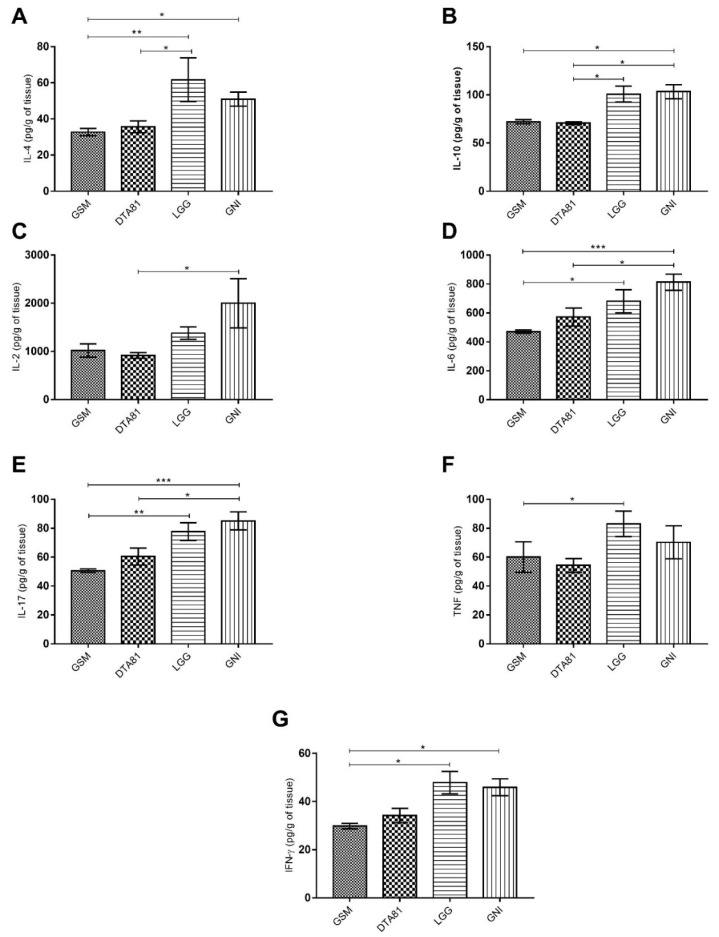
Pro- and anti-inflammatory cytokine profile in colon tissue using the cytometric bead array kit. (**A**) Interleukin-4 (IL-4); (**B**) IL-10; (**C**) IL-2; (**D**) IL-6, (**E**) IL-17; (**F**) tumor necrosis factor (TNF); (**G**) interferon-gamma (IFN-γ). Values are expressed as means ± SEM (*n* = 6). *, *p* ≤ 0.05; **, *p* ≤ 0.01; ***, *p* ≤ 0.001.

In total, the levels of seven cytokines (IFN-γ, IL-2, IL-4, IL-6, IL-10, IL-17, and TNF) were measured in colon tissue by flow cytometry (Figure 3). A remarkable low level of pro- and anti-inflammatory cytokines were observed in GSM and DTA81 groups, whereas the highest level was noticed in the GNI group. Animals receiving DTA81 displayed lower amounts of the anti-inflammatory cytokines interleukin IL-4 (Figure 3A) and IL-10 (Figure 3B) in colonic homogenates compared to the LGG group. Group DTA81 showed also lower levels of pro-inflammatory cytokines IL-2 (Figure 3C), IL-6 (Figure 3D), and IL-17 (Figure 3E) compared to the GNI group. Lastly, the levels of the pro-inflammatory cytokines TNF and IFN-γ were significantly higher in the LGG group when compared to the control group GSM (Figure 3F,G).

### 3.4. The Caecal Concentration of SCFA

**Figure 4:**
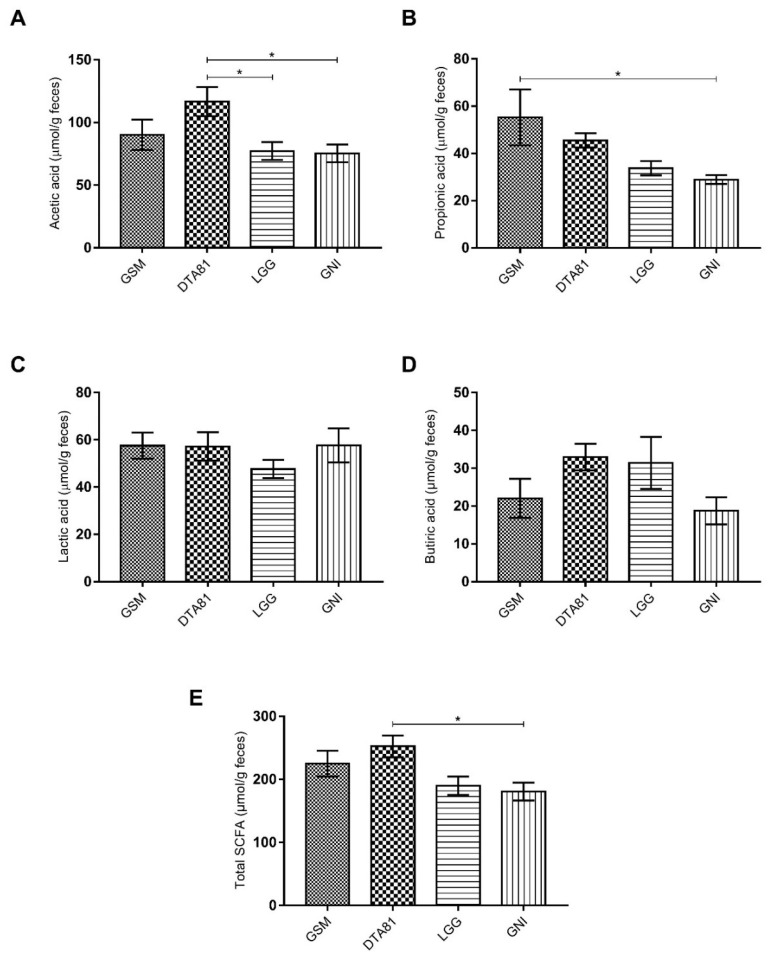
Caecal concentration of short-chain fatty acids (SCFAs). (**A**) Acetic acid; (**B**) propionic acid; (**C**) lactic acid; (**D**) butyric acid; (**E**) total SCFA. Values are means ± SEM (*n* = 6). *, *p* ≤ 0.05.

The SCFA profile, namely acetate, propionate, lactate, and butyrate were evaluated in fecal samples collected from the caecum at the end of the experimental period and the results are shown in Figure 4. The level of acetic acid was higher in group DTA81 (Figure 4A) compared to LGG (*p* = 0.046) and GNI (*p* = 0.034) groups, whereas a higher propionic acid concentration was only observed in the GSM group when compared to the GNI group (Figure 4B, *p* = 0.038). There was no statistical difference in the levels of lactate and butyrate among the groups (Figure 4C,D, *p* > 0.05), although on average a higher amount of butyric acid can be verified in the groups DTA81 (32.95 ± 3.5) and LGG (31.4 ± 6.9) when compared to GSM (22.03 ± 5.17) and GNI (18.73 ± 3.57). Lastly, the total production of SCFA, represented by the sum of acetate, propionate, lactate, and butyrate, was significantly higher in the group DTA81 when compared to the group GNI (Figure 4E, *p* = 0.027).

### 3.5. RT-qPCR

**Figure 5:**
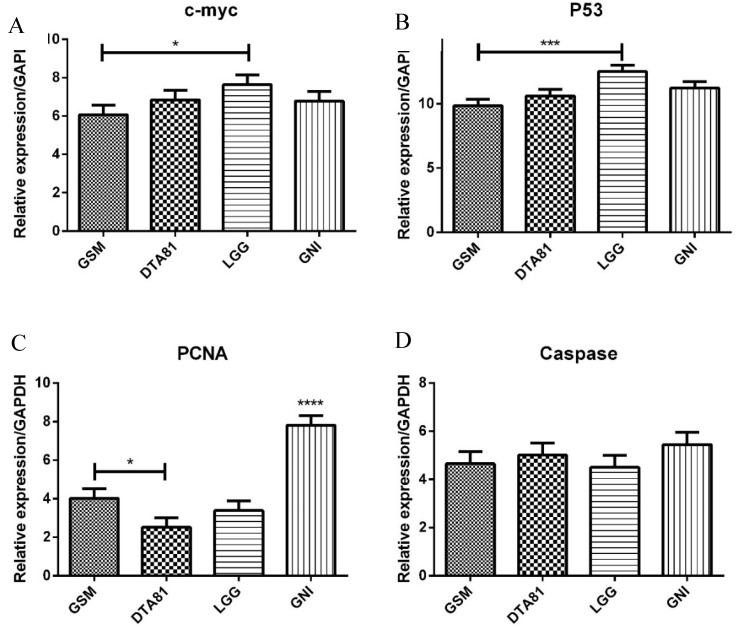
Gene expression assessment by quantitative real-time polymerase chain reaction (RT-qPCR) of four tumor-related genes associated with CRC development. (**A**) *c-myc*; (**B**) *p53*; (**C**) PCNA; (**D**) Caspase-3. Values are means ± SEM (*n* = 6). *, *p* ≤ 0.05; ***, *p* ≤ 0.001; ****, *p* ≤ 0.0001.

The quantitative PCR analysis showed the effect of different interventions on the expression of four important genes. In half of the genes analyzed (*c-myc* and *p53*), the LGG group has comparable behavior, statistically higher expression when compared to skim milk control (GSM). The proliferating cell nuclear antigen (PCNA) gene expression was statistically lower for all treated groups relatively to GNI, with DTA81 presenting the lower value (statistically lower than GSM). Caspase-3 expression showed no statistical differences among the groups (Figure 5).

### 3.6. Bacterial Community Profile

#### 3.6.1. Alpha and Beta Diversity Analyses

The bacterial composition before (*t*0) and after (*t*1) the experimental period was investigated to study the effect of oral administration of *L. paracasei* DTA81 and *L. rhamnosus GG* on the gut bacterial community structure and abundance of relevant taxa during the development of precancerous mucosal lesions induced by the carcinogenic DMH.

Following high throughput amplicon sequencing of the hypervariable region V1-V2 of the 16S rRNA genes, a total of 1,277,498 high-quality sequences with an average length of 496 bp from the 32 fecal samples (mean: 39,922 ± 11,299) were obtained after the removal of low quality and chimeric sequences. Rarefaction curves evidenced that sequencing depth was enough to capture the majority of bacterial phylotypes from all samples analyzed.

**Figure 6:**
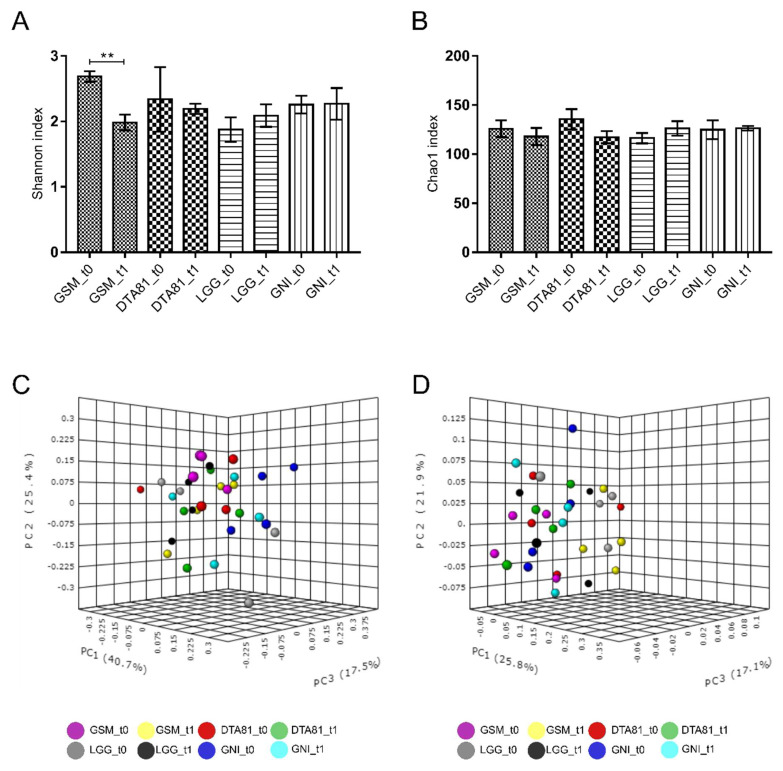
Bar charts comparing species diversity (**A**) and richness (**B**) between the different groups (GSM, DTA81, LGG, and GNI) before (*t*0) and after (*t*1) the experimental period. Values are means ± SEM (*n* = 4). **, *p* ≤ 0.01. Principal coordinate analysis (PCoA) based on weighted (**C**) and unweighted (**D**) UniFrac distances for GSM, DTA81, LGG, and GNI in the time-points *t*0 and *t*1. Permutational multivariate analysis of variance (PERMANOVA) was carried out to detect significant dissimilarities among the different experimental groups.

The Shannon diversity index (Figure 6A), which accounts for microbial richness and evenness, varied significantly only inside the group GSM, whereas no significant differences were observed for the Chao1 richness index among experimental groups (Figure 6B). To evaluate whether *L. rhamnosus* GG and *L. paracasei* DTA81 significantly shifted gut bacterial community structure, a PERMANOVA test was performed on both the weighted and unweighted UniFrac matrices. As reported in Figure 6C,D, samples are largely separated in both weighted and unweighted UniFrac PCoA plots, explaining 83.6 and 64.8% of the total variance, respectively. Our analysis indicates a low dissimilarity among the four different groups at *t*0 and *t*1 (PERMANOVA weighted UniFrac: *F*-value = 1.71, R^2^ = 0.34, *p* < 0.019; PERMANOVA unweighted UniFrac: *F*-value = 1.39, R^2^ = 0.29, *p* < 0.061).

#### 3.6.2. Microbiota Profiling and Linear Discriminant Analysis Effect Size (LEfSe) Analysis

With the aim to identify differences in microbiota composition at different taxonomic levels associated with early CRC development and probiotic administration (microbial biomarkers), a LEfSe was conducted.

**Figure 7:**
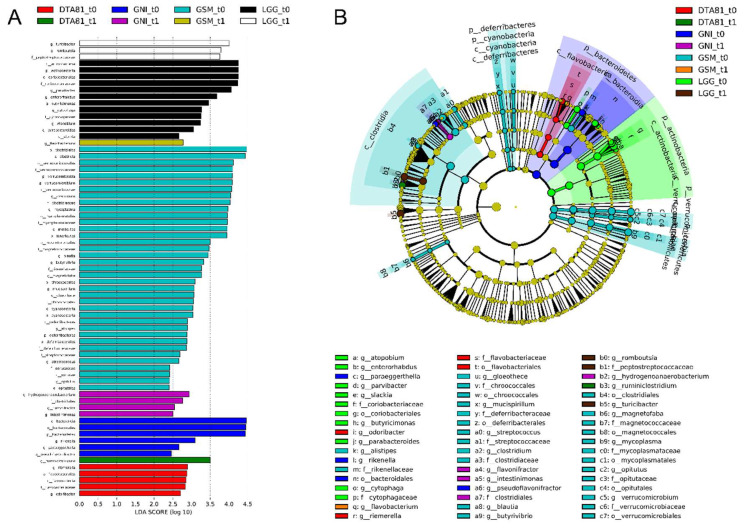
Linear discriminant analysis effect size (LEfSe) analysis to identify significant differences in abundant taxa (biomarkers) before (*t*0) and after (*t*1) the different interventions. (**A**) Taxonomic groups showing linear discriminant analysis (LDA) scores > 2.0 with false discovery rate (FDR) *p* < 0.05. Letters: *p*, phylum; c, class; o, order; f, family; g, genus. (**B**) Cladogram showing taxa with different abundance among the groups (LDA score > 2.0, FDR *p* < 0.05). Letters have the same equivalence to those in parentheses in (**A**).

After the clustering process at a genetic distance of 3%, a total of 541 OTUs were obtained following data filtering based on the removal of low count (3394 OTUs removed) and low variance (61 OTUs discarded) features. The number of significantly discriminative biomarkers identified by 16S rRNA gene amplicon sequencing is depicted in Figure 7 and was determined employing the LEfSe tool.

A total of 69 significant features were identified as biomarkers that most likely explained microbiome shifts between the adaptation phase (*t*0) and at the end of the experimental period (*t*1). This analysis reveals a remarkable depletion in the majority of taxa after the experimental period across all groups, but a relevant reduction was specifically observed in the group GSM_*t*0 encompassing 5 different phyla (*Verrucomicrobia*, *Tenericutes*, *Cyanobacteria*, *Deferribacteres,* and *Bacteroidetes*).

After the probiotic intervention, an enrichment of the genus *Ruminiclostridium* (LDA score >3.0) was observed in the DTA81 group, while the genera *Romboutsia* and *Turicibacter*, both with LDA scores greater than 3.5, were found as biomarkers in the LGG group. With regard to the groups that did not undergo interventions with probiotics, GNI_*t*0 displayed higher counts (LDA score >2.5) of the genera *Hydrogenoanaerobacterium*, *Flavonifractor,* and *Intestinimonas*. Lastly, a significant enrichment of *Flavobacterium* (LDA score >2.5) in the GSM_*t*1 group was noticed.

## 4. Discussion

It is well established that *lactobacilli* administration can reduce the risk of colorectal cancer development or be used as an adjuvant treatment during anticancer chemotherapy [10,35,36]. In this study, we evaluated the chemopreventive effects of ingestion of *Lactobacillus paracasei* DTA81 on the initial stages that precede the development of pre-neoplastic lesions in DMH-induced early colon carcinogenesis (CRC) in mice. The use of this strain reduced liver oxidative stress and gut inflammation, along with positively modulated gut microbiota and increased the total production of SCFAs.

Following eight weeks of the experimental period, no significant differences in terms of body weight and food consumption were noticed among all groups, indicating that probiotic intervention did not interfere with weight gain. However, a significant and reduced food consumption on the days after DMH injection was observed in all experimental groups, which is a common adverse effect observed for this drug [37]. Overall, the procarcinogen DMH needs a series of metabolic reactions to become active. The main stages occur in the liver where DMH is converted to methylazoxymethanol, which in turn is conjugated with glucuronic acid and secreted through the bile duct gaining access to the intestine [38]. Finally, specific bacterial taxa that display β-glucuronidase activity (e.g., *Bacteroides* spp., *Eubacterium* spp., and *Clostridium* spp.) can hydrolyze the complex previously formed in the liver with the consequent release of azoxymethane, an active carcinogenic compound, capable of activating colon carcinogenesis [39].

Oxidative stress is fundamentally the result of an imbalance between reactive oxygen and nitrogen species (ROS-RNS) production and antioxidants’ capability to scavenge cytotoxic compounds [4]. As mentioned, the liver has a detrimental role in DMH activation, which results in excessive ROS production. Besides, increased oxidative stress in the liver may also reflect changes in the intestinal barrier [40]. More specifically in terms of CRC development, chronic colonic inflammation, or environmental stressors lead to a condition of oxidative stress that should be reduced in the precancerous stage as an anticancer response strategy [41]. In this context, several studies have reported that probiotics supplementation can improve gut antioxidant status through different mechanisms and, consequently, to prevent preneoplastic lesions formation [42]. In this study, we verified a relevant antioxidant activity of *L. rhamnosus* GG, which occurred by a significantly reduced hepatic level of lipid peroxidation marker (malondialdehyde–MDA) and protein carbonyl content associated with less demand for the enzymes involved in the endogenous antioxidant system (SOD and CAT). In a comparable manner, *L. paracasei* DTA81 was able to reduce on average the levels of liver TBARs and protein carbonyl, but without statistical significance when compared to the control groups. Evaluating the effects of the strain *L. paracasei* M5L on human HT-29 cells, Hu et al. (2015) [43] observed reduced cellular activity of SOD and CAT following lactobacilli treatment. Conducting a meta-analysis of 23 studies involving different types of oxidative stress models, Zhao et al. (2020) [44] noticed that probiotics can significantly increase hepatic levels of SOD, GSH-PX, and reduce MDA content. Similar outcomes are reported after probiotic or even metabiotic intervention during DMH-induced colon carcinogenesis in animal models. Walia et al. (2018) [45] observed low activity levels of the enzymes GSH, GPx, GST, SOD, and catalase in colon tissue of rats after DMH treatment, however, the administration of *Lactobacillus plantarum* (AdF10) combined or not with *L. rhamnosus* GG for 16 weeks was able to increase the activity of both antioxidant enzymes. Evaluating the effects of the administration of metabiotics extracted from probiotic *L. rhamnosus* MD 14, Sharma and Shukla (2020) [46] reported enhanced antioxidant levels of SOD, GSH, and GPx associated with a reduced lipid oxidation level.

The continuous production and secretion of pro-inflammatory cytokines such as IL-6, IL-17, and TNF have been associated with human colonic carcinogenesis [47]. According to Lamichhane et al. (2020) [48], probiotics administration can effectively modulate immune functions in the gut by reducing the production of tumor-promoting cytokines. The present study showed that the highest concentrations of the pro-inflammatory cytokines IL-6 and IL-17 were found in the colon tissue of animals subjected to DMH treatment only (GNI group), whereas the intervention with the probiotic candidate *L. paracasei* DTA81 reduced significantly the levels of both cytokines. Milk intake also promoted a beneficial anti-inflammatory effect in the colon, which is in consonance with previously published studies [49,50,51,52,53,54]. It is well established that IL-6 and its overexpression has a detrimental role in early CRC tumorigenesis by promoting tumor growth and invasion [55]. Furthermore, IL-6 acts as one of the main molecules able to trigger Th17 cell differentiation and stabilization [56]. As reported elsewhere, high concentrations of IL-17 have been detected in the sera and tumor tissues of CRC patients, playing an important role in CRC tumorigenesis and metastasis [57,58,59]. Moreover, IL-17 secreting CD4^+^ T cells infiltrated in colorectal tumors can coproduce TNF-α and IL-10 [60]. Comparably with the previous study, a higher level of both cytokines (Il-17, TNF-α, and IL-10) was observed in colonic homogenates of the animals which have not undergone any intervention (GNI group). Surprisingly, *L. rhamnosus* GG appears among the strains with an anti-inflammatory activity using a single or a mixed formulation [61,62,63,64]. However, our results show a lower anti-inflammatory capacity of this isolate compared to the probiotic candidate *L. paracasei* DTA81. A possible explanation could be the relatively short experimental period adopted in this study. Generally, the beneficial effects of LGG in animal models for CRC have been observed after a long-term period of probiotic administration (ranging from 12 to 16 weeks) [45,65]. In terms of DTA81, a possible mechanism for such a low bowel inflammatory status could be due to the strong adhesion capability of this isolate, higher than *L. rhamnosus* GG, evaluated in HT-29 human cancer cells [19], combined with its modulatory effect favoring the growth of short-chain fatty acid-producing bacteria.

Produced by specific intestinal commensal bacteria through the fermentation of dietary fibers, short-chain fatty acids (SCFAs) are health-promoting bioactive molecules with anti-inflammatory and anticarcinogenic properties acting by the control of regulatory mechanisms such as suppression of nuclear transcription factor kappa B (NF-κB) and antiproliferative activity [66,67]. Our results show that the daily intake of *L. paracasei* DTA81 significantly increased the caecal level of acetate and total SCFAs (the sum of acetate, propionate, lactate, and butyrate) when compared to the group GNI. As a matter of fact, acetate can also be converted to butyrate, and both molecules are utilized as an energy source for colonocytes. As reported by several studies [68,69,70], it is assumed that reductions around 1 µg/L of acetate and butyrate in human stools can increase the risk of colon cancer and adenoma development by 84.2% and 71.3%, respectively. Moreover, a reduction of both molecules can be positively associated with colorectal tumorigenesis [71,72]. Although lactic acid bacteria are unable to produce some SCFAs, such as butyrate, probiotic strains can modulate the composition of the intestinal microbiota through different mechanisms and favor the growth of SCFA-producing bacteria. Ferrario et al. (2014) [73] showed that the intake of *L. paracasei* DG impacted positively on the microbiota and short-chain fatty acids of healthy volunteers with low initial butyrate concentrations (<25 mmol/kg of wet feces), contributing greatly to an increment of this organic acid (329 ± 255%). As discussed below, *L. paracasei* DTA81 ingestion favored the growth of *Ruminiclostridium* spp., an SCFA-producing bacterium.

The modulation of gene expression involved in cell proliferation, differentiation, and apoptosis is one of the mechanisms suggested to be responsible for the antitumor effect of probiotics [16,74] or their metabolites, as assumed for *L. paracasei* NTU 101 fermented skim milk extracts [75]. The *c-myc* oncogene belongs to a family of genes that regulate cell growth, proliferation, differentiation, cell cycle progression, metabolism, survival, and apoptosis. Its dysregulation is involved in genomic instability and carcinogenesis [76,77]. In the present study, an increased *c-myc* expression was observed in the colonic tissue of the animals that received LGG when compared to the GSM group, but not between DTA81 and GNI groups. As demonstrated in previous studies [78,79], *c-myc* expression is triggered following DMH injection, and, specifically regarding the LGG group, the harmful carcinogen effect was promptly counteracted by *p53* activation, which is considered a classic tumor suppressor gene, activated in response to oncogenic or other cellular stresses. It is responsible for regulating other genes involved in cell-cycle arrest, DNA repair, senescence, and apoptosis [80]. A high frequency of sequential inactivation of tumor suppressor genes, including TP53 and APC, is observed in colorectal cancer [81]. In the present study, an increase in *p53* expression was observed in the LGG group compared to the GSM group, which is in agreement with previous results evidenced in animals induced by colorectal carcinogenesis with DMH [65].

It is suggested that the genus *Lactobacillus* can induce apoptosis of tumor cells by activating *p53* and pro-apoptotic proteins, such as Caspase 3 [82]. Although Caspase 3 levels did not differ among the experimental groups, additional pro-apoptotic genes, such as *Bax* and Caspase 9, may also contribute to the balance between proliferation and apoptosis of tumor cells. With regard to *L. paracasei* strains and apoptosis’ regulation, their activities are attributed to different mechanisms such as by stimulation of proapoptotic genes [48], the promotion of cell cycle arrest, and calreticulin translocation via the generation of ROS, observed for *L. paracasei* M5L [43], and by modulating the expression of specific Bcl-2 family proteins as hypothesized to the strain *L. paracasei* K5 [83]. Immunomodulation is another possible mechanism that contributes to the proapoptotic activity induced by probiotics, especially increased TNF production [67], as observed in the present study for the LGG group. Lastly, we also investigated the expression levels of the PCNA, which were significantly reduced in the intervention groups. The PCNA is a non-histamine nuclear protein that participates in DNA replication, along with the reconstruction of the double DNA strands, and is considered a marker of cell cycle kinetics and proliferative activity. PCNA levels are directly correlated with malignancy and tumor invasion, vascular infiltration, and survival [84,85]. The ability of probiotics, especially *Lactobacillus acidophilus*, *Lactobacillus salivarius* Ren (LS), *Bifidobacterium bifidum*, and *Bifidobacterium lactis* to suppress PCNA expression has been demonstrated in experimental studies [86,87,88].

In recent years, gut microbiota composition has been associated with the initiation and progression of CRC. Specifically, dysbiosis is frequently associated with inflammatory bowel disorders and several types of cancer [89,90]. Therefore, understanding of the general composition of the gut microbiota in CRC and the development of novel strategies for the prevention and treatment of this disease are needed. In this scenario, the administration of specific probiotic strains has been demonstrated to be a valuable prophylactic or therapeutic option to manage gut dysbiosis in several diseases [91,92,93,94].

With regards to alpha and beta diversity analyses, here assessed employing Shannon/Chao1 indices and PERMANOVA on the weighted/unweighted UniFrac matrices, the intra-group analysis revealed a reduced Shannon index only within of the GSM group. Overall, a lower microbial diversity in fecal samples of people with CRC is noticed when compared with the healthy controls [95,96], although Sheng et al. (2019) [97] and Hibberd et al. (2017) [98] have reported greater alpha diversity in patients with CRC in comparison with healthy subjects. In terms of bacterial community structure, a slight shift was observed in all groups when weighted UniFrac distance was considered (*p* < 0.05), since samples from the different groups were not clustered separately from each other, which may due to a transient state in dysbiosis formation in the untreated control groups GSM and GNI [98].

In order to further identify differences in the microbiota composition, specifically microbial biomarkers associated with early CRC development and probiotic administration, a linear discriminant analysis effect size (LEfSe) was carried out. Our results revealed the presence of CRC biomarkers enriched in the untreated group GNI, i.e., not undergone probiotic administration, but not in the control group that received skim milk. *Hydrogenoanaerobacterium* is a proteolytic bacterium able to degrade sulfur-containing amino acids and producing sulfide [99], which can be a genotoxic and pro-inflammatory compound [100]. With regards to *Intestinimonas*, this is a bacterial genus present in the intestinal tract of human and other animals with the capability to produce butyrate from sugars and amino acids [101]. Notwithstanding this fact, a lower concentration of butyric acid was observed in the caecal content of the animals belonging to the GNI group when compared to the intervention groups. According to Sheng et al. (2019) [97], *Hydrogenoanaerobacterium* and *Intestinimonas* were detected in fecal samples of patients with stage II and stage III CRC, correspondingly. Notwithstanding the absence of a consensus about the role of *Flavonifractor* in CRC, also detected in higher abundances in healthy colonic samples than in CRC ones [102], a recent study conducted by Gupta et al. (2019) [103] revealed an association between CRC and *Flavonifractor plautii* in Indian patients due to the capability of this microorganism to degrade beneficial anticarcinogenic flavonoids.

Taking into consideration probiotic administration, after LGG administration for 8 weeks LEfSe analysis enabled the identification of two putative microbial biomarkers during early experimental colon carcinogenesis. According to Mangifesta et al. (2018) [95], the genus *Romboutsia* is frequently associated with a healthy status of patients, and its reduction in intestinal mucosal injuries carcinogenic mucosa/adenomatous polyps is correlated with early tumor formation. The prophylactic treatment with LGG also increased the abundance of the genus *Turicibacter* in fecal samples. In pigs pretreated with a low-dose of a probiotic mix (*Bacillus licheniformis* and *Bacillus subtilis*), the expansion of *Turicibacter* population, as well as *Clostridium* and *Lactobacillus*, was associated with host defense against enteropathogenic *Escherichia coli* [104]. Conversely, *Turicibacter* abundance was also reduced in dogs with IBD, however, administration of a mix of eight different probiotic strains could not restore this taxon to the initial levels [105]. Overall, *L. rhamnosus* GG was able to positively modulate the intestinal microbiota, although the presence of specific taxa associated with the production of anti-inflammatory or pro-apoptotic compounds were not observed, which may justify the weak anti-inflammatory activity and the low SCFA content found in the gut of the animals.

*L. paracasei* DTA81 intake remarkably increased the abundance of the genus *Ruminiclostridium*, an SCFA-producing bacteria member of the *Firmicutes* phylum, which can be associated with the higher acetate and total SCFA production observed in caecal samples of the DTA81 group. It is worth mentioning that, in this study, the abundance of acetic acid bacteria (family *Acetobacteraceae*, genera *Gluconobacter* and *Acetobacter*) were found to be lower than 0.5% throughout the groups (established threshold to data filtering). Notwithstanding this fact, we identified a higher abundance of the genus *Allobaculum* (Log 2 fold change: DTA81 = 9.6; GSM = 1.1; LGG = 0.7; GNI = 0.8) after DTA81 treatment, although without statistical significance (FDR *p* > 0.05). As described elsewhere [106,107,108], *Allobaculum* is considered a beneficial microorganism with a putative capability to protect intestinal barrier function by producing short-chain fatty acids, particularly acetate. In terms of CRC development, a higher abundance of *Ruminiclostridium* may contribute to reducing colitis-associated cancer, as reported by Zhang et al. (2018) [109] in an AOM/DSS murine model. Also noteworthy this taxon appears with an increased abundance (LDA score > 3.0) after that a mix of probiotics, composed of *Bifidobacterium longum*, *Lactobacillus acidophilus*, *Enterococcus faecalis*, was offered for 9 weeks to mice in a model of colitis-associated cancer [110]. *Ruminiclostridium* also arises as a putative biomarker in healthy colonic mucosa samples when compared to polyp-associated tissue, usually found in lower abundance [95,111,112].

## 5. Conclusions

The administration of *L. paracasei* DTA81 strain can reduce liver lipid peroxidation and PCNA expression levels, ameliorate gut immunological status, positively modulate the intestinal microbiota by increasing the abundance of *Ruminiclostridium*, and increase the caecal levels of acetic acid and total SCFA production. Therefore, our results indicate that *L. paracasei* DTA81 can mitigate the precedent stages involved in CRC development, although additional investigations are needed adopting a long-term study with a larger cohort to evaluate further biomarkers of colon carcinogenesis such as aberrant crypt foci (ACF) formation and related pathways such as Apc/β-Catenin and K-Ras.

## Figures and Tables

**Table 1:** Table of primer sequences of four genes associated with colorectal cancer (CRC) development.

Gene	Sequence (5’→3’)	Reference
PCNA	F: TAAAGAAGAGGAGGCGGTAA R: TAAGTGTCCCATGTCAGCAA	[28]
Caspase-3	F: AGCAGCTTTGTGTGTGTGATTCTAA R: AGTTTCGGCTTTCCAGTCAGAC	[29]
*c-myc*	F: TCCTGTACCTCGTCCGATTC R: GGAGGACAGCAGCGAGTC	[30]
*p53*	F: GTATTTCACCCTCAAGATCC R: TGGGCATCCTTTAACTCTA	[31]
GAPDH	F: CTGCTTCACCACCTTCTTGA R: AAGGTCATCCCAGAGCTAAA	[32]

## Supplementary Materials

The following are available online at https://www.mdpi.com/2076-2607/8/12/1994/s1, Figure S1: Animals were randomized by body weight before starting the experimental period. There were no statistically significant differences among the groups (*p* > 0.05). Figure S2. Food intake comparison among the groups following DMH injection was carried out with the non-parametric Kruskal-Wallis method and Dunn’s post-hoc multiple comparison test. Data represent the mean weight during the experimental period. Data are shown as mean ± SEM.
