# Supplementary material for: Chemoprevention of DMH-Induced Early Colon Carcinogenesis in Male BALB/c Mice by Administration of Lactobacillus Paracasei DTA81
Source: Microorganisms. 2020 Dec 14;8(12):1994. doi: 10.3390/microorganisms8121994 (PMC7765108; doi:10.3390/microorganisms8121994)
Supplement: Supplementary file 1 [file microorganisms-08-01994-s001.pdf]

Chemoprevention of DMH-Induced Early Colon Carcinogenesis in Male BALB/c Mice by Administration of *Lactobacillus Paracasei* DTA81

Vinícius da Silva Duarte [1,2], Bruna Cristina dos Santos Cruz [3], Armin Tarrah [1], Roberto Sousa Dias [4], Luiza de Paula Dias Moreira [1], Wilson José Fernandes Lemos Junior [5], Lívia Carneiro Fidélis Silva [2], Gabriele Rocha Santana [4], Leandro Licursi de Oliveira [4], Maria do Carmo Gouveia Peluzio [3], Hilario Cuquetto Mantovani [2], Viviana Corich [1] and Alessio Giacomini [1,*] and Sérgio Oliveira de Paula [4,*]

1 Department of Agronomy Food Natural Resources Animals and Environment, University of Padova, Viale dell'Universitá, 16, 35020 Legnaro (PD), Italy; vinicius.dasilvaduarte@unipd.it (V.d.S.D.); tarrah.armin@gmail.com (A.T.); luiza.depauladiasmoreira@studenti.unipd.it (L.d.P.D.M.); viviana.corich@unipd.it (V.C.)

2 Department of Microbiology, Av. Peter Henry Rolfs, s/n, Campus Universitário, Universidade Federal de Vicosa, 36570-900 Vicosa, Brazil; livia.silva@ufv.br (L.C.F.S.); hcm6@ufv.br (H.C.M.)

3 Department of Nutrition and Health, Av. Peter Henry Rolfs, s/n, Campus Universitário, Universidade Federal de Vicosa, 36570-900 Vicosa, Brazil; brunacruz09@yahoo.com.br (B.C.d.S.C.); mcgpeluzio@gmail.com (M.d.C.G.P.)

4 Department of General Biology, Av. Peter Henry Rolfs, s/n, Campus Universitario, Universidade Federal de Vicosa, 36570-900 Vicosa, Brazil; roberto.dias@ufv.br (R.S.D.); gabi.rocha-s@hotmail.com (G.R.S.); leandro.licursi@ufv.br (L.L.d.O.)

5 Faculty of Science and Technology, Free University of Bolzano-Bozen, 39100 Bolzano, Italy; juniorjflemos@gmail.com

* Correspondence: alessio.giacomini@unipd.it (A.G.); depaula@ufv.br (S.O.d.P.); Tel.: +39-328-0390077 (A.G.); +55-31-3612-5016 (S.O.d.P.)

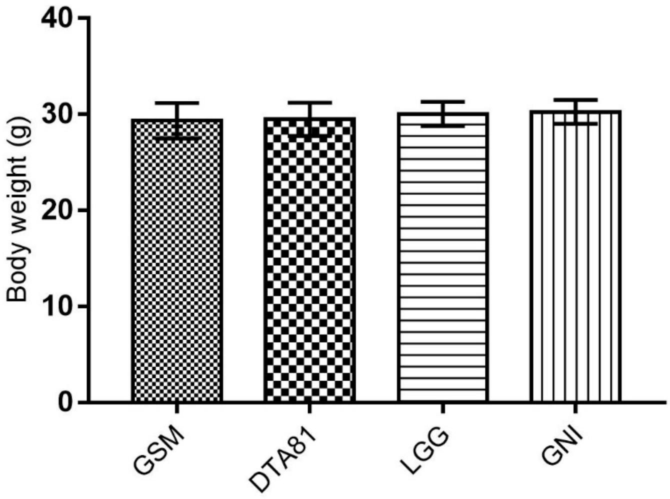

Figure S1. Animals were randomized by body weight before starting the experimental period. There were no statistically significant differences among the groups ($p > 0.05$).

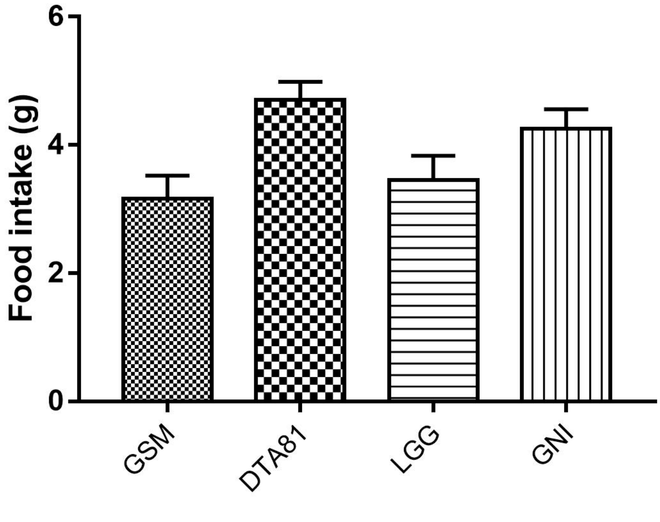

Figure S2. Food intake comparison among the groups following DMH injection was carried out with the non-parametric Kruskal-Wallis method and Dunn's post-hoc multiple comparison test. Data represent the mean weight during the experimental period. Data are shown as mean ± SEM.
